# Spatial Immunometabolism: Integrating Technologies to Decode Cellular Metabolism in Tissues

**DOI:** 10.1002/eji.70094

**Published:** 2025-11-14

**Authors:** Felix J. Hartmann

**Affiliations:** ^1^ German Cancer Research Center (DKFZ) Systems Immunology & Single‐Cell Biology Heidelberg Germany; ^2^ German Cancer Consortium (DKTK) DKFZ Core Center Heidelberg Germany

**Keywords:** antitumor immunity, cellular metabolism, immunometabolism, multiplexed imaging, spatial biology, systems immunology, tumor microenvironment

## Abstract

The metabolic programs of immune cells influence their activation, differentiation, and effector functions. While much of immunometabolism has focused on cell‐intrinsic regulation, it is now clear that metabolic activity is profoundly influenced by the surrounding tissue environment. In tumors and other inflammatory settings, immune cells are shaped by nutrient gradients, hypoxia, and immunoregulatory metabolites, factors that are spatially heterogeneous and often poorly captured by traditional methods. This review highlights recent technological advances that enable spatially resolved analysis of immune metabolism, with an emphasis on multimodal integration and cancer as a model system. Mass spectrometry imaging (MALDI, DESI), high‐resolution platforms like SIMS, and vibrational imaging approaches such as Raman microscopy enable direct visualization of metabolites in tissue. Transcriptomic and proteomic data can be used to infer metabolic states, and computational models are being developed to integrate these diverse data layers. Together, these technologies are transforming the study of immunometabolism from dissociated cells to the intact tissue context. Key challenges remain in resolution, annotation, and data integration, but spatial immunometabolism holds particular promise for illuminating mechanisms of immune regulation in health and disease.

## Graphical abstract

This review highlights recent advances that enable spatially resolved analysis of immunometabolism within tissue microenvironments. Integrating mass spectrometry imaging, vibrational microscopy, and spatial omics reveals how metabolic organization shapes immune function in cancer and other pathologies.

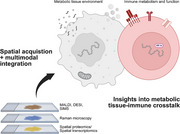

## 1 Immunometabolism in Spatial Context

The metabolic programs of immune cells are tightly linked to their function, phenotype, and fate. While foundational studies in immunometabolism have revealed how metabolic pathways support immune activation, differentiation, and memory formation [1], it is increasingly evident that these programs are not solely cell‐intrinsic but shaped by the surrounding tissue environment [2].

**FIGURE 1:**
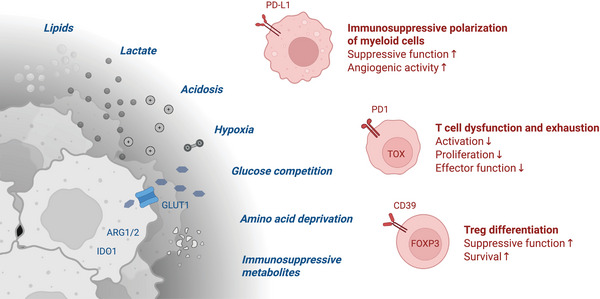
Spatially organized metabolic constraints and immune cell adaptations in the tumor microenvironment. Local metabolic conditions within the tumor microenvironment (TME) differentially regulate various immune cells. Often, the tumor core is characterized by hypoxia, acidosis, and depletion of glucose and amino acids, resulting in accumulation of immunosuppressive metabolites such as lactate, adenosine, and kynurenine. These metabolic features drive T cell exhaustion, support regulatory T cells, and polarize macrophages toward immunosuppressive, lipid‐rich states. In contrast, vascularized regions are more permissive to nutrient uptake and support metabolically active immune cells with enhanced effector function. Together, this spatial organization creates distinct metabolic niches that constrain or support antitumor immunity, highlighting the need for spatially resolved technologies to uncover these local interactions.

As immune cells migrate through the body, they encounter metabolically diverse environments characterized by nutrient gradients, hypoxia, and pH fluctuations. These features create distinct metabolic niches that can reinforce or suppress immune function in a location‐specific manner. In the tumor microenvironment (TME), glucose and amino acid depletion, hypoxia‐induced lactate accumulation, and lipid remodeling can contribute to immune dysfunction (Figure 1) [3]. Lactate is a prototypical example of an immunoregulatory metabolite: it acts not only as an alternative fuel but also as an epigenetic regulator and suppressor of effector T cell function [4]. More broadly, metabolites such as adenosine, kynurenine, itaconate, and succinate serve as local immunomodulators by engaging specific receptors or intracellular sensors in immune cells [5]. Lipid metabolism is another key axis of immune regulation. Fatty acid oxidation supports long‐lived regulatory T cells, while lipid droplet accumulation has been linked to macrophage polarization and dendritic cell function [6]. Yet the spatial organization and cellular interactions of these lipid‐driven programs within tissues are still poorly understood. Stromal cells, including cancer‐associated fibroblasts and adipocytes, further modulate immune responses through both nutrient competition and secretion of immunosuppressive metabolites [7].

To resolve these complex and spatially confined immunometabolic programs, new technologies are needed [8, 9]. While novel approaches are moving toward the quantification of the metabolome of individual cells [10, 11], many still obscure the spatial organization of metabolic environments, masking critical tissue heterogeneity. A deeper understanding of spatially organized metabolic programs could reveal new mechanisms of immune evasion, identify metabolic vulnerabilities, and guide precision immunotherapy. This review takes a method‐driven approach to highlight recent technological advances that are transforming the study of spatial immunometabolism, with a particular focus on multimodal integration and its application in the context of cancer biology. Different mass spectrometry imaging (MSI) platforms and other spatial omics are examined in terms of how they have been adapted and combined to uncover new biological insights. Rather than treating each modality in isolation, the review emphasizes how their convergence enables a deeper understanding of immune metabolism in situ. The article concludes with a perspective on emerging concepts, computational challenges, and future opportunities for this rapidly evolving field.

## 2 Multimodal Imaging Technologies Transform Immunometabolism

### 2.1 Mass Spectrometry Imaging: From Metabolites to Immune Niches

To uncover how metabolic programs are spatially organized within tissues, researchers have long relied on imaging approaches that directly visualize metabolites in situ (Table 1). Among these, MSI provides a strategy to map the biochemical composition of tissue environments without requiring specific probes [12]. This section introduces how MSI is used to determine the metabolic tissue environment and highlights how integration with immune phenotyping and spatial transcriptomics has revealed the functional impact of metabolic remodeling in the TME.

**TABLE 1:** Comparison of technologies used in spatial immunometabolism.

Method	Spatial resolution	Molecules measured	Throughput	Phenotyping	Key limitations
MALDI	Multicellular (5–50 µm)	Lipids, metabolites (matrix‐dependent)	Medium	No	Moderate resolution; molecular identification complex; limited detection of small polar metabolites
DESI	Multicellular (30–100 µm)	Lipids, small metabolites	Medium	No	Lower spatial resolution; fewer lipids detected; molecular identification complex
SIMS	Subcellular (<1 µm)	Lipids, small metabolites, fragments	Low	No	Low chemical ID confidence; complex instrumentation; limited coverage of larger metabolites
Raman	Subcellular (0.3–5 µm)	Lipids, proteins, labeled metabolites	Low	Indirect (vibrational signatures)	Low sensitivity for small molecules; limited multiplexing; high technical cost
Spatial proteomics	Subcellular (0.3–5 µm)	Proteins (including enzymes, transporters)	High	Yes	Requires validated antibody panels; does not directly measure metabolites
Spatial transcriptomics	Cellular (1–100 µm technology‐ dependent)	mRNA (including metabolic genes)	High	Yes	Inference only; low abundance of metabolic transcripts; trade‐off between resolution and gene coverage

*Note*: Each platform varies in its analyte coverage, spatial resolution, and capacity for immune phenotyping. Spatial resolutions given are commonly reported values and can vary with the technical setup. Integration of multiple modalities is often required to connect metabolite distributions with immune cell identity and function.

**FIGURE 2:**
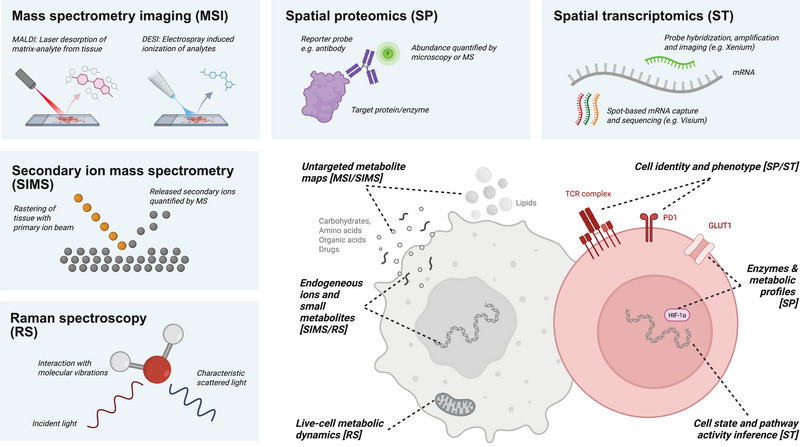
Technological toolbox for spatial immunometabolism. MALDI and DESI mass spectrometry imaging (MSI) ionize analytes directly from tissue sections for subsequent mass spectrometric analysis. These approaches generate untargeted maps of metabolites and lipids at near‐single‐cell resolution, enabling the identification of metabolic gradients and nutrient niches across tissue architecture. In contrast, SIMS employs a focused primary ion beam to sputter molecular fragments from the tissue surface. With its submicron resolution and sensitivity for small metabolites and lipids, SIMS is particularly powerful for subcellular analyses. Vibrational microscopy, exemplified by Raman spectroscopy (RS), exploits the interaction of incident photons with molecular vibrations to produce characteristic spectral fingerprints. RS enables label‐free visualization of metabolic processes such as lipid turnover and glucose uptake in live or fixed samples. Antibody‐based spatial proteomics (SP) platforms use tagged antibodies to detect proteins or posttranslational modifications in situ, with readouts obtained via fluorescent tags (e.g., Codex) or metal isotopes (e.g., IMC, MIBI). These methods complement MSI by identifying cell types, profiling metabolic enzymes and transporters, and linking immune function to local metabolism. Spatial transcriptomics (ST) captures mRNA molecules either through spatially barcoded arrays followed by sequencing (e.g., Visium) or by in situ hybridization with complementary probes (e.g., Xenium). Coupled with computational tools, ST enables inference of metabolic pathway activity and cellular states. Integrating these complementary technologies provides a powerful means to connect metabolic environments with cellular remodeling, illuminating how metabolic heterogeneity and cell–cell crosstalk regulate immune function in situ.

In MSI, tissues are analyzed pixel‐by‐pixel, and the mass‐to‐charge (m/z) ratios, which uniquely identify molecules by how they behave in an electric field, are analyzed at each location (Figure 2). The most widely used MSI platform is matrix‐assisted laser desorption/ionization (MALDI), which has become foundational for exploring metabolic heterogeneity within tissues [13]. Its strength lies in its ability to map a broad spectrum of biomolecules, particularly low molecular weight molecules such as lipids, metabolites, or drugs, directly from intact tissue sections without the need for labeling or prior knowledge of the exact targets. Besides MALDI, desorption electrospray ionization (DESI) presents another ionization technique for MSI studies. DESI does not require matrix deposition, which can interfere with the measurement of low molecular weight species, thus making DESI‐MSI particularly suited for the quantification of small metabolites. Tumor sections and other tissues have been metabolically analyzed with MALDI and DESI for a number of years, revealing the heterogeneous distribution of nutrients and metabolites within these complex environments [14]. However, studies of immune cell metabolism are scarce so far, and likely attributed to the fact that its spatial resolution has long not been single‐cell resolving. Typically, MALDI has 5–50 µm and DESI 30–100 µm spatial resolution, although some groups have achieved around 1 µm for MALDI [15, 16] and below 10 µm for DESI [17, 18] using sophisticated set‐ups. In addition to resolution, MALDI‐based immune cell studies have been limited by the fact that MSI lacks intrinsic means for cell‐type identification and phenotyping.

To address these restrictions, researchers interested in immunometabolism and its microenvironmental regulation have started to integrate MSI with other layers of analysis [19]. Employed strategies range from simple immunohistochemistry or immunofluorescence to high‐dimensional spatial proteomic or transcriptomic approaches. These combinations are synergistic: MSI provides untargeted maps of metabolite and lipid distributions, while complementary spatial omics layers identify and spatially locate cell subsets based on gene/protein expression and phenotype. Together, they enable direct correlation between local metabolic environments and the phenotypic identity or functional state of immune cells.

Early proof‐of‐concept work integrated MALDI with multiplexed immunofluorescence on sequential tissue sections [20, 21]. These pioneering efforts laid the experimental groundwork for multimodal co‐registration and used metabolic‐proteomic overlays to map intratumoral drug metabolism [21] or to highlight that macrophages adapt phenotypically to their nutrient environments [20]. This enabled recent studies integrating MALDI and spatial proteomics through imaging mass cytometry (IMC) on the same colorectal tumor tissue sections [22]. Analyzing the identical section allowed direct co‐registration of metabolic maps with high‐dimensional phenotyping of individual cells. With this, the researchers identified a spatially confined subset of CD204‐expressing tumor‐associated macrophages (TAM) enriched in specific glycerophospholipids. These lipid signatures suggested a distinct metabolic adaptation within a functionally specialized TAM subset, demonstrating the power of this multi‐omic approach to reveal immune‐metabolic niches in situ.

Besides proteomics, MSI has recently been integrated with spatial transcriptomics platforms to complement the metabolic information with single‐cell expression profiles. For example, a recent study employed the 10× Visium technology and MALDI to study human glioblastoma samples [23]. Focusing on malignant cells, the authors identified hypoxic tumor regions across both datasets that corresponded to different genetic tumor subclones, thus revealing how cancer genetics and metabolism are spatially interconnected. However, given the spot‐based nature of Visium, which does not reach single‐cell spatial resolution, the authors did not focus on immune cells and their regulation. Another study combined DESI (specifically its airflow‐assisted variation AFADESI) and MALDI with spatial transcriptomics to map metabolite distributions in gastric cancer [24]. AFADESI was used to identify amino acids, polyamines, and nucleotides, while MALDI characterized lipid distributions. Integration of spatial transcriptomics allowed region‐specific correlation of metabolic and transcriptional profiles. Together, this revealed heterogeneous arginine and proline metabolism, as well as a coordinated spatial shift in lipid and glutamine usage in B cells, depending on proximity to the tumor boundary. While these early studies were mostly measured on subsequent tissue slides and thus had to focus on larger immune cell aggregates, Vicari et al. [25] recently presented a protocol that allows Visium and MALDI on the same section.

MSI has also been extended to support functional metabolic analysis via stable isotope tracing. Introducing 13C‐labeled substrates such as glucose into cell culture or animal models has allowed downstream detection of isotope incorporation into metabolic products [26]. Since these early studies did not reach single‐cell resolution and relied solely on MSI, they mostly focused on profiling healthy or malignant mouse brain tissues. Building on this, a recent approach termed 13C‐SpaceM has coupled MALDI isotope tracing with fluorescence microscopy and image‐based cell segmentation to infer metabolic flux into fatty acids at near‐single‐cell resolution [27]. In glioma models, 13C‐SpaceM has revealed heterogeneous fatty acid synthesis within the tumor, offering a dynamic perspective on tissue metabolism that static abundance profiling cannot provide, but stopped short of investigating immune cell metabolism in the TME.

Together, these studies illustrate how spatial immunometabolism studies are increasingly possible through the combination of MALDI and DESI metabolomics and single‐cell resolved spatial phenotyping. Despite these exciting developments, such multi‐omics studies still face several technical challenges. For one, small water‐soluble metabolites such as sugars, amino acids, and organic acids remain difficult to detect. This is because these molecules ionize poorly in mass spectrometry, especially when other compounds interfere, a phenomenon known as ion suppression. This leads to biased coverage toward metabolites that ionize efficiently, which is illustrated by the focus on fatty acid and lipid metabolism, especially across MALDI‐based spatial immunometabolism studies. Related, metabolite identification, especially for low‐mass metabolites, is often ambiguous in the absence of tandem MS or validated spectral libraries. Nevertheless, MSI is evolving from a standalone imaging tool into a key component of multimodal tissue cartography. The integration of MALDI and DESI into spatial immunometabolism studies offers a critical window into the biochemical landscape of the tissue that surrounds and shapes immune cell behavior and allows researchers to link metabolic niches with immune cell phenotypes.

### 2.2 SIMS‐Based Platforms: From Subcellular Landscapes to Cellular Competition

While MALDI and DESI provide wide chemical coverage, their spatial resolution has historically limited cellular‐level analysis. By contrast, secondary ion mass spectrometry (SIMS) offers submicron resolution and excels in uncovering fine‐grained spatial structure. SIMS uses a focused ion beam to sputter secondary ions directly from the tissue surface, achieving spatial resolution down to the nanometer scale. Though traditionally used in materials science, SIMS has recently been adapted for spatial immunometabolism, enabling analysis of single cells and even subcellular compartments within tissues [28].

An example of this potential is SEAM (spatial single nuclear metabolomics), developed to resolve metabolite signatures at the nuclear level [29]. Applying time‐of‐flight SIMS to fibrotic liver tissue, the authors generated high‐resolution maps of endogenous ions and small metabolites within individual nuclei. These nuclear signatures could distinguish cell types, including human Kupffer cells, based on their subcellular metabolic profiles, offering a new layer of classification beyond transcriptomic or proteomic identity. SEAM illustrates how SIMS enables exploration of metabolically regulated cellular states at spatial scales inaccessible to other modalities. In a separate study, SIMS has also been extended to 3D imaging. An approach termed 3D‐SMF (three‐dimensional spatial metabolic fingerprinting) stacks multiple SIMS images from serial tissue sections to reconstruct 3D metabolic maps [30]. 3D‐SMF was applied to human tonsil tissue and combined with heavy‐metal labelled antibodies that could be read out directly by SIMS to define immune compartments. This approach provided a spatially resolved view of metabolic tissue architecture and highlighted how specific lipid distributions correlate with immune microenvironments such as T cell–rich or stromal regions. Building on these ideas, Hu et al. [31] introduced scSpaMet, a technique that integrates high‐resolution SIMS with IMC‐based spatial proteomics. This allowed the team to create joint maps of cell phenotype and metabolic content from the same sample. Applied to lung tumors and human tonsil, scSpaMet revealed metabolically defined immune cell subsets and spatially organized T cell states, including metabolically distinct zones of CD4^+^ and CD8^+^ T cells. Extending this further, a recently published protocol combines SIMS, fluorescence and electron microscopy with tracing of heavy isotope labelled nutrients into a single pipeline which could allow identification of individual immune cells and quantification of their nutrient uptake in the context of different tissue environments [32].

Taken together, these studies illustrate the power of SIMS to resolve subcellular and single‐cell metabolic features with high spatial precision. Each study highlights a different dimension of what SIMS can contribute: from nuclear‐level profiling to 3D spatial mapping to single‐cell multi‐omics. However, these advances also underscore key limitations. While SIMS provides structural detail, it often lacks molecular identification for many fragments. In addition, it requires high‐cost, sophisticated instrumentation and computational infrastructure for image acquisition and interpretation. Nevertheless, SIMS has carved out a distinctive niche within spatial immunometabolism with its capacity for high‐resolution, label‐free imaging. Especially when integrated with proteomic or optical modalities, SIMS is uniquely suited for probing the metabolic state of individual immune cells in their native environment.

## 3 Inferring Immune Metabolism from Genes and Proteins

As outlined above, MSI technologies such as MALDI, DESI, and SIMS enable untargeted detection of metabolites directly from tissue. However, applying this at scale across large clinical cohorts remains challenging, particularly when high resolution or throughput is required. To overcome these constraints, recent studies have explored how other spatial omics layers, such as protein and gene expression, can be used to infer metabolic information by quantifying components of the cellular metabolic network.

For example, antibody‐based single‐cell and spatial proteomics were used to analyze the metabolic profiles of individual immune cell types [33, 34, 35]. These studies quantified metabolic regulators such as nutrient transporters, metabolic enzymes, signaling molecules, and transcription factors that together orchestrate metabolic programs. Importantly, these approaches do not measure metabolites or flux directly, but instead capture the expression of proteins that govern metabolism to provide a proxy for cellular metabolic capacity. From these proteomics profiles, pathway‐level features such as glycolytic or oxidative ability can be computed. These features have been validated against traditional metabolic readouts, such as extracellular flux analysis and others [33, 36, 37, 38, 39, 40]. Combining this antibody‐based concept with the MIBI (multiplexed ion beam imaging) spatial proteomics technology revealed that CD8^+^ T cells with a metabolically exhausted phenotype preferentially localize to large intratumoral aggregates, spatially separated from the tumor edge [33]. Conversely, metabolically active subsets were enriched near the tumor‐immune border.

Antibody‐based approaches provide different opportunities compared with metabolite imaging. They provide single‐cell resolution and are compatible with archived, formalin‐fixed tissue, where proteins are more stable than small molecules. Together with their throughput, these platforms thus enable large‐scale profiling. On the other hand, these methods require prior selection of target proteins and careful experimental design to cover relevant metabolic pathways [9]. Moreover, antibody validation remains critical to ensure specificity and avoid misleading results.

Transcriptomic data, including single‐cell and bulk RNA‐seq, have also been used to infer metabolic activity, using various strategies [41, 42, 43, 44, 45]. One set of methods includes tools such as AUCell [46], single‐cell gene set enrichment analysis (scGSEA) [47], and scMetabolism [48]. These tools typically compute pathway scores based on the expression of metabolic genes, providing an estimate of a cell's transcriptional potential for processes like glycolysis or fatty acid oxidation. While straightforward to apply, such scores may not capture functional relationships between enzymes and are sensitive to data sparsity. More sophisticated approaches use constraint‐based modeling, most prominently, flux balance analysis (FBA), to simulate the flow of metabolites through a cell's metabolic network. These models use the known inputs and outputs of biochemical reactions, along with physical limits like energy usage, to simulate which metabolic pathways are likely active in a given cell. Notable implementations of such concepts are CellFie [49, 50], Compass [51], and METAFlux [52]. Machine learning–based models, such as scFEA [53] and the MINN framework [54], integrate gene expression with network logic to predict pathway usage at single‐cell resolution.

Some of these concepts have been extended to spatial transcriptomics, particularly spot‐based technologies. For example, Liu et al. [55] analyzed oral squamous cell carcinoma and identified metabolically distinct tumor regions enriched for regulatory T cells and fibroblasts in hypermetabolic zones. This supports the notion that spatial gene expression patterns can reflect local metabolic environments, despite providing proxies rather than direct readouts. Similarly, Yang et al. applied single‐cell and spatial transcriptomics to clear cell renal cell carcinoma, revealing spatially organized metabolic programs across tumor and immune compartments [56]. CD8⁺ T cells with high energy metabolism signatures also exhibited early exhaustion markers, and M2‐like macrophages were enriched in sphingolipid metabolism. Their computational tool, scMet, enabled reconstruction of single‐cell metabolic profiles from bulk RNA‐seq, expanding the applicability of metabolic inference and linking metabolic states to clinical outcome.

Despite rapid progress, several challenges remain. Many computational approaches have not been comprehensively benchmarked against experimental flux measurements. Instead, validation has been limited to a few exemplary metabolic pathways. Further, spatial transcriptomics adds complexity in comparison to single‐cell analyses: typical spatial sequencing‐based platforms such as Visium offer genome‐wide coverage but lack single‐cell resolution, while probe‐based methods such as Xenium require predefined panels and may omit key metabolic enzymes. Novel developments, such as Visium HD [57], could fill this gap, combining improved spatial resolution with untargeted readouts. So far, these have not been employed to comprehensively study cellular metabolism. Overall, low transcript abundance makes it difficult to detect subtle differences in metabolic capacity. Improving resolution and sensitivity, as well as integrating these tools with single‐cell datasets, will thus be crucial for advancing transcription‐based spatial immunometabolism research.

Taken together, advances in both transcriptomic and proteomic technologies are rapidly improving our ability to map immune metabolism in tissues and provide an orthogonal layer to metabolite measurements. Spatial proteomics methods especially excel at the high‐throughput analysis of a large number of clinical tissue sections, thus providing a novel lens to quantify immune cell metabolism and its impact on therapeutic outcomes. Spatial transcriptomics, together with computational modeling, enables broad inference of metabolic programs and their relation to cell state. Together, these tools promise to reshape our understanding of immune regulation in tissues and support the development of metabolism‐informed diagnostics and therapies.

## 4 Vibrational Microscopy: Dynamic Views of Immune Metabolism

Besides the established MSI technologies and the new possibilities of inferring metabolic profiles from other omics layers, there are exciting developments in spectroscopy‐based technologies that can be used to study immunometabolism [58]. A prime example is Raman scattering, an optical phenomenon in which incident photons interact with molecular vibrations [59]. Each molecule produces a characteristic Raman spectrum, a so‐called molecular fingerprint, that can be used to infer its identity and abundance. This provides the foundation for Raman microscopy, a powerful imaging technique capable of visualizing the spatial distribution of biomolecules such as lipids, proteins, and small metabolites directly in cells and tissues. In addition, probes can be added to analyze additional specific molecules [60]. Since Raman microscopy does not rely on fluorescent labels or fixation, it is capable of live‐cell imaging, opening the door to studying immune cell metabolism in real time within physiologically relevant environments. For example, Raman microscopy has been used to trace glucose uptake using alkyne‐tagged glucose analogs [61], monitor lipid synthesis via deuterium incorporation [62], and visualize lipid droplet dynamics during inflammation [63]. Imaging speeds are fast enough to capture dynamic processes, including immune cell motility, polarization, or metabolic shifts upon stimulation.

Importantly for spatial immunometabolism, Raman microscopy offers single‐cell to subcellular resolution. It is possible to detect metabolic heterogeneity among immune cells based on differential accumulation of lipids or altered protein synthesis. Immune cells undergoing metabolic reprogramming often display distinct vibrational signatures, such as elevated C–H stretching from lipid biosynthesis or deuterium‐labeled bonds from new macromolecule formation. These can be visualized within intact lymph nodes, tumors, and in vivo [64], enabling researchers to link metabolic state with anatomical localization and cellular phenotype.

Despite its promise, the technology has some limitations. While label‐free Raman imaging excels at detecting abundant biomolecules like lipids and proteins, its sensitivity for low‐abundance small metabolites is lower than that of mass spectrometry. Multiplexing is also more challenging; most Raman applications focus on a few spectral windows unless hyperspectral imaging and computational unmixing are used. Moreover, access to advanced Raman platforms is still limited by their cost and technical complexity, although commercial systems are becoming more accessible. Nevertheless, the potential is substantial. Raman microscopy has already been employed in cancer research to image lipid droplet remodeling in cancer cells [65] and could be extended to investigate immune‐tumor metabolic crosstalk, nutrient competition, or spatially restricted immunosuppressive niches. With the growing availability of bioorthogonal vibrational probes and advances in computational spectral unmixing, it is now feasible to monitor discrete metabolic pathways in live immune cells within their native environments. In sum, Raman microscopy bridges the resolution of fluorescence microscopy, the chemical specificity of mass spectrometry, and the live‐cell compatibility of metabolic tracing, positioning it as a key enabler for future discoveries in spatial immunometabolism.

## 5 Where We Go From Here: Outlook on Spatial Immunometabolism

This review has outlined how recent advances in MSI technology, spatial omics, and vibrational imaging have enabled in situ analysis of immune metabolism at increasing depth and resolution. Together, these tools now allow researchers to investigate how immune cells adapt to tissue‐specific metabolic constraints, uncovering spatially organized programs that influence function and fate.

Researchers looking to enter this exciting field have to consider several factors when deciding which technologies to employ (Figure 2). Depending on the question, whether identifying metabolic niches, tracing nutrient competition, or mapping immunosuppressive environments, different platforms offer complementary advantages (Table 1). MSI methods like MALDI and, more recently, DESI, are providing a well‐established, untargeted opportunity to reveal the metabolic tissue environment. While routine implementations have not provided the single‐cell spatial resolution necessary to analyze individual immune cells, novel approaches such as MALDI‐2 are making this increasingly possible [16]. Complementing this data on the metabolic environment, spatial proteomics and transcriptomics, with their single‐cell resolution and high‐multiplexing ability, are uniquely suited to identify and profile the functional and phenotypic states of immune cells. To directly quantify immune cell metabolism, SIMS offers a high‐resolution MS‐based opportunity where instrumentation is available.

Across all MS platforms, metabolite annotation from complex spectra remains a challenge. Despite being untargeted, sample preparation and machine setups greatly influence the classes of metabolites detected by mass spectrometry, and we are still far from achieving comprehensive, one‐shot quantification of the entire metabolome with about 40000 metabolites [66]. Emerging tools such as nanoDESI, which allow matrix‐free sampling with improved sensitivity for small polar metabolites [67], could help expand the detectable metabolome in spatial immunometabolism studies. This may enable better coverage of key immune‐regulatory molecules like amino acids, nucleotides, and organic acids, presenting a unique opportunity for spatial immunometabolic studies.

As in many other fields, a remaining challenge pertains to the data analysis of spatial multi‐omics studies in general [68]. These studies produce large and complex datasets with different biological axes of variation. Factor analysis [69], neural networks [70], and incorporating prior biological knowledge [71] present potential avenues for deriving biological insights from these complex datasets.

Given these challenges, most studies to date have focused on technological improvements and presented illustrative examples of how this data could lead to fundamental biological insights and clinical impact. Most of the metabolism‐centered multi‐omics studies provided proof‐of‐concept in a limited set of samples but did not analyze large cohorts of clinical samples to relate their findings to patient outlook. This might be related to challenges in high‐throughput sample processing and acquisition in multi‐omics, but also to the fact that metabolites are often lost in tissues that have been fixed and stored for long periods, for example, in FFPE format. This is where deducing metabolic states from proteomic or transcriptomic data provides an opportunity. Spatial transcriptomics provides high coverage of the genetic network across many metabolic pathways, and high‐resolution sequencing is poised to become more broadly accessible [72]. Metabolic proteins and enzymes directly carry well‐known metabolic conversions and are, by themselves, potential therapeutic targets [73], but require the establishment of targeted panels in advance. Immunologists should be familiar with these technologies, and both approaches can be readily applied to large clinical sample cohorts to, for example, identify metabolic changes during therapy and activation, as well as identify metabolic states associated with therapeutic success.

Although most spatial immunometabolism studies to date focus on cancer, similar principles apply to nonmalignant contexts. Chronic infections [74, 75, 76], autoimmune diseases [51, 77, 78], and tissue homeostasis [79, 80, 81] are all characterized by immunometabolic heterogeneity that is spatially organized and shaped by the local microenvironment. Future studies may leverage spatial multi‐omics to uncover immunoregulatory metabolic circuits across these diverse disease settings.

Overall, I believe that with these most recent developments, we are now ready to bring immunological research into context with the cell's metabolic environment. Making use of animal models and genetic targeting, we can figure out the metabolic determinants of tumor–immune interactions. Employing spatial multi‐omics and machine learning provides an avenue to characterize the metabolic makeup of tissue sections together with the tissue's immunological phenotype and link these layers directly in human patient material, offering a powerful path toward understanding and overcoming treatment resistance, and opening new frontiers in how to modulate immunity in the tissue environment.

## Conflicts of Interest

The author declares no conflicts of interest.

## Peer Review

The peer review history for this article is available at https://doi.org/10.1002/eji.70094.

## Declaration of AI‐Assisted Technologies

Generative A.I. tools were used to improve the structure and readability of the manuscript. After using these services, F.J.H. reviewed and edited the content as needed and takes full responsibility for the content of the publication.

## Data Availability

Data sharing does not apply to this article as no datasets were generated or analyzed during the current study.
